# Assessing the Effects of Habitat Loss and Deterioration on a Red Squirrel Translocation Site: Insights for Future Conservation Management

**DOI:** 10.1002/ece3.70482

**Published:** 2024-10-25

**Authors:** Emily Reilly, Colin Lawton

**Affiliations:** ^1^ Zoology, School of Natural Sciences, Ryan Institute University of Galway Galway Ireland

**Keywords:** carrying capacity, clear‐felling, feeding survey, forest fire, habitat availability, *Sciurus vulgaris*, translocation

## Abstract

Translocations, a conservation tool used to conserve and restore dwindling species, are often associated with high failure rates. Inadequate long‐term monitoring of both populations and their introduction sites beyond the initial years post‐translocation creates a gap in our understanding of the factors that determine translocation success or failure, resulting in less informed projects in the future. This lack of long‐term monitoring is partly caused by the absence of a well‐defined framework by which the success of the translocation can be measured, leading to premature and sometimes inaccurate assessments of their outcome. We investigated the long‐term outcome of a red squirrel translocation in the west of Ireland, specifically assessing the habitat changes in the translocation site since the introduction in 2005, and their impact on the capacity of the forest to sustain a population of a given size. Using digitised historical map data, we showed that the translocation site experienced a 53% reduction in suitable habitat. Additionally, there was a 41%–81% reduction in the total number of red squirrels the forest could support, according to feeding survey data. Clear‐felling, a forest fire and a shift in tree species composition collectively contributed to this decline in site suitability. This investigation underscores the complexity of translocation projects and emphasises the pivotal role of habitat quality in their outcomes. We advocate for detailed habitat assessments during the planning phase, avoidance of unstable habitats as translocation sites, and the implementation of long‐term monitoring practices.

## Abstract 2

Translocations often face high failure rates, due in part to our lack of understanding of their long‐term outcomes. We investigated long‐term outcome of a red squirrel translocation in the west of Ireland, focusing on habitat loss and changes in carrying capacity. We found a 53% reduction in suitable habitat and a 41%–81% decline in carrying capacity, emphasising the importance of detailed habitat assessments and the use of stable sites for successful translocations.

## 1 Introduction

Translocations, the deliberate movement of organisms from one location to another (IUCN/SSC 2013), are an effective conservation tool used to recover populations and reduce extinction rates (Berger‐Tal, Blumstein, and Swaisgood 2020). However, translocations are inherently risky and prone to failure (Fischer and Lindenmayer 2000), mainly due to their low foundation population numbers (Verbeylen, De Bruyn, and Matthysen 2003) and isolation (Fahrig and Merriam 1985), making them susceptible to extinction. Generally, the success of a translocation is measured by the establishment of a self‐sustaining population (Fischer and Lindenmayer 2000). However, this definition is controversial as it can only indicate success at the time of assessment and does not necessarily indicate the long‐term viability of the population (Seddon 1999). A translocation project can be divided into three distinct phases: the establishment phase, the growth phase and the regulation phase (IUCN/SSC 2013). We suggest that the success of the project is measured by the population's successful transition through these various stages. The duration of each stage is dependent on the life history traits of the target species, but the success of the stage may be determined by the same general outcomes (Seddon 1999).

The establishment phase begins with the release of animals to the site and may be considered to be successful if the initial translocation group survives the translocation process and remains in the new habitat. The period immediately following release poses considerable challenges, with high mortality rates within the first few weeks (Calvete and Estrada 2004), mainly due to predation (Calvete and Estrada 2004; Davis 1983; Metzgab 1967) and an increased vulnerability due to handling and relocation stress (Dickens, Delehanty, and Michael Romero 2010). Homing behaviours and hyperdispersal, which may lead to the departure of part or the entire founding population, also pose serious threats to the success of a translocation (Bilby and Moseby 2023; Guilbert et al. 2007; Jones, Archer, and Dickman 2003; Short and Turner 2000; Steen 1994). If the population successfully survives these challenges it will enter the growth phase (IUCN/SSC 2013). This phase is characterised by the successful reproduction of the founding population and their offspring (Seddon 1999), and expansion of their range (IUCN/SSC 2013). However, numerous factors can impede this growth, including predation (Moseby et al. 2011; Plein et al. 2016), competition for resources (Danielson and Gaines 1987; Losos and Spiller 1999) and an unsuitable habitat (Blyton et al. 2023; Nafus et al. 2017). Additionally, the failure to remove the original issue prompting the translocation (Fischer and Lindenmayer 2000; Wilson 2018) and social structure disruption (Shier 2006; Shier and Swaisgood 2012) can also hinder population expansion and survival during the growth phase. While these factors may not directly cause mortality in the founding population, their cumulative negative effects may become apparent over time, affecting the overall success of the translocation. Typically, monitoring of translocation projects does not continue beyond the growth phase. The final phase of a translocation, known as the regulation phase (IUCN/SSC 2013), is characterised by the continued presence of the population and a stabilised population density. Threats such as inbreeding depression, disease, human disturbance and habitat‐related factors such as habitat loss and deterioration in habitat quality may all impact the success of the translocation in the long‐term (IUCN/SSC 2013).

Threats to the success of the regulation phase are generally cumulative over time, and do not have immediate effects on the population. Long‐term monitoring is required to reveal the extent of these effects on the population, and reveal the true success of the translocation. For example, the effects of habitat‐related factors may be delayed, leading to a decline in the population years after the habitat change (Lira, de Souza Leite, and Metzger 2019). For translocated individuals, the translocation process itself induces a change in their habitat, meaning that delayed responses in the population to their new environment should be expected in the years following their introduction. Habitat‐related factors play a crucial role in the success or failure of translocation efforts (Berger‐Tal, Blumstein, and Swaisgood 2020; Bubac et al. 2019). Habitat loss, often cited as the reason for initiating a translocation project (Griffith et al. 1989; Seddon 2010), can also be a primary factor contributing to its failure in the long term (Bubac et al. 2019). Habitat loss has several negative consequences for a population including a reduced carrying capacity (Devore 2014), disruptions in the social structure (Verbeylen et al. 2009), heightened predation risks (Lawrence 1966; Leahy et al. 2016), fragmentation (Stratford and Robinson 2005), and reduced food availability (Merrick et al. 2021). Habitat loss has detrimental effects on small mammal abundance (Engstrom 2010; Johnstone, Lill, and Reina 2014; Legge et al. 2008), with some species exhibiting weak to no recovery capabilities in the years following the initial habitat loss (Pardon et al. 2003). Habitat specialists, such as the red squirrel (*Sciurus vulgaris*) are particularly vulnerable to habitat loss (Ellis and Coppins 2007; Wauters et al. 1994).

Poor habitat quality, whether pre‐existing or as a result of deterioration, is another leading cause of translocation failures (Bellis et al. 2019; Griffith et al. 1989; Wolf et al. 1996). The suitability of a habitat depends on characteristics such as habitat structure, composition, and resource availability, in combination with the specific requirements of the target species. Unsuitable habitats may negatively impact reproductive output (Harig and Fausch 2002), survival (Blyton et al. 2023; Nafus et al. 2017), and population density (Johnson et al. 2018; Moorhouse, Gelling, and Macdonald 2009). For the red squirrel, food availability is the primary factor influencing population densities (Wauters et al. 2008) and is crucial in determining reproductive output (Rodrigues et al. 2010). Consequently, a decline in the quality of the food source could have severe implications for the population's overall health and long‐term viability.

The translocation of 19 red squirrels to Derryclare forest was conducted in 2005 in an effort to conserve the Irish red squirrel population, which experienced a dramatic decline in the years following the grey squirrel introduction to Ireland in 1911. This translocation followed, and was informed by, several other red squirrel translocations in Europe which were deemed successful, at least in the short‐term (Dennis et al. 2011; Fornasari, Casale, and Wauters 1997; Shuttleworth 2010; Shuttleworth, Kenward, and Jackson 2009). The Derryclare site was chosen based on its size, age structure and tree composition (Poole and Lawton 2009). Additionally, the region is free from the presence of the invasive grey squirrel (*Sciurus carolinensis*), the underlying cause for the decline in the red squirrel population in Ireland (Teangana et al. 2000). The future of the translocated population was predicted during the planning stage. Specifically, a population viability analysis was conducted, accounting for the effects of inbreeding depression, and the mean probability of extinction after 20 years was estimated at 0.125 (Poole 2007). Monitoring by Poole and Lawton (2009) from 2005 to 2007 revealed the success of the translocation through the establishment phase, indicated by the population's initial survival and continued presence. Subsequent monitoring of the translocated population was conducted by Waters (2012) from 2008 to 2012. This study revealed that the population was continuing to develop through the growth phase, with an estimated population of 51, as determined through live trapping, and an expansion of their range into new areas within the forest.

In the years following the introduction of the red squirrels, the habitat underwent several changes, some of which were unforeseen. Expected and unexpected clear‐felling operations occurred multiple times in the coniferous section of Derryclare from 2005 onwards, due to its commercial nature. Additionally, the forest suffered from an unexpected and significant fire in 2011. Although no direct squirrel mortalities were found to be caused by the fire or felling (Waters 2012), the long‐term effects on the habitat and subsequent impact on the red squirrel population, including any possible delayed response to these combined habitat losses, remain unknown.

The aim of our study was to investigate the current habitat available to the population, and determine the impact of clear‐felling and fire on the potential carrying capacity for red squirrels in the forest. Specifically, we quantified the area lost since 2005 by digitising historical map data of Derryclare and comparing it to the current available habitat. To investigate changes in the carrying capacity of the population within the forest and variations in actual population size since the translocation, we conducted a feeding survey. The results of our feeding survey were then compared to feeding survey data from 2008 to 2011, allowing us to track the available and consumed energy for all surveyed years. An additional estimate of the carrying capacity of red squirrels in the forest was calculated based on the available forest area, for the year of the initial translocation and the years of the feeding surveys. We hypothesise that there has been a loss of available suitable habitat through clear‐felling and fire, and that this in turn has reduced the red squirrel carrying capacity of the population in the forest. We also predict that this habitat loss will impact the success of the translocation.

## 2 Methods

### 2.1 Study Site

Derryclare commercial forest is a 570‐ha forest located in Connemara, Co. Galway, situated at the foot of Mount Derryclare, one of The Twelve Bens. The forest was planted in the 1960s and is managed by the state‐owned commercial forestry business, Coillte. It primarily comprises two coniferous species, lodgepole pine (*Pinus contorta*) and Sitka spruce (*Picea sitchensis*). Other species present in Derryclare include heather (*Calluna vulgaris*), Japanese Larch (*Larix kaempferi*) and moor grass (*Molinia caerulea*). According to Coillte data reported in Waters (2012), 31% of the forest is bare or felled, due to its commercial nature. Adjacent to the commercial forest is a 19‐ha nature reserve owned by the National Parks and Wildlife Service. Within the nature reserve, 13 ha comprise mature broadleaf forest, consisting primarily of oak (*Quercus petraea*), ash (*Fraxinus excelsior*), birch (*Betula* sp.) and hazel (*Corylus avellana*). The remaining area consists of young birch plantations and wetland.

### 2.2 Investigating Extent of Habitat Loss

The composition of Derryclare has undergone significant changes since the introduction of red squirrels to the forest in 2005. To quantify these changes, maps from the establishment phase monitoring project (Poole and Lawton 2009) and growth phase monitoring project (Waters 2012), which were based on Coillte data, were digitised using QGIS. This process involved overlaying the old maps on a recent map containing forest stand borders and reclassifying the stands according to the old maps. The resulting maps detailed the habitat composition of Derryclare for the years 2005 and 2008–2011. Precise composition data for other years was not available. The composition of Derryclare in 2021 was mapped on QGIS using recent Coillte data and validated through on‐site observations.

For each digital map, forest stands were classified as either mature forest, immature forest, bare forest, or other species (refer to ‘Study site’). Mature forest was categorised as any stand consisting of lodgepole pine or Sitka spruce aged 25 years or older, as this is the age at which stands begin producing a good cone crop (Gurnell, Lurz, and Pepper 2001). Stands below this age were classified as immature. Immature stands cannot sustain a red squirrel population, although can still facilitate movement through the habitat (Gurnell, Lurz, and Pepper 2001).

### 2.3 Feeding Surveys

**FIGURE 1:**
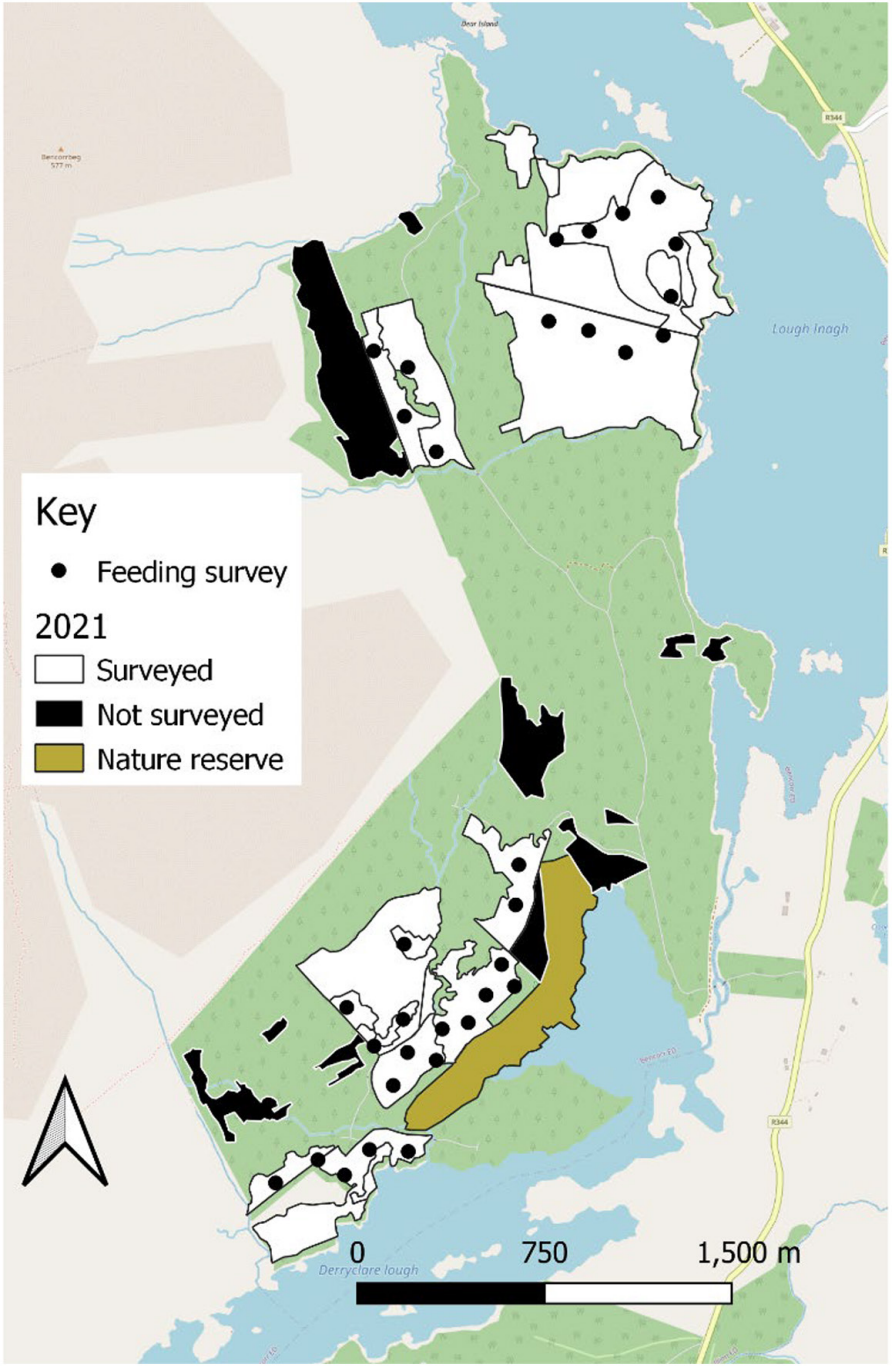
Derryclare Forest, depicting mature forest stands that were surveyed (white) and not surveyed (black) in 2021 and feeding survey transect locations (black dots). Mature forest that was not surveyed was inaccessible. The nature reserve was not suitable for conifer cone surveys. The surrounding forest (light green) either consisted of bare or immature forest.

Feeding survey data from the growth phase monitoring project (2008–2011) were obtained from Waters (2012). Additional feeding surveys were subsequently conducted by the authors of the current study in October 2021 to assess the carrying capacity of the red squirrel population in Derryclare and estimate the actual red squirrel population size. During these surveys, cones were counted and collected from the ground along 33 transect lines, each measuring 50 m in length and 1 m in width. Surveys encompassed all mature stands, as immature, bare and felled areas cannot support red squirrel populations. A small proportion of the mature forest was inaccessible, and therefore not surveyed, as illustrated in Figure 1. The starting points of all transects were located at least 100 m apart.

During the surveys, all cones (unconsumed and consumed) were identified by tree species and counted. All consumed cones, identified by their obvious stripping of cone scales, were collected, as were a sample of unconsumed cones from each block (approximately 5–10 unconsumed cones per transect of each tree species, depending on availability). Unconsumed cones were collected for the purpose of estimating seed numbers.

#### 2.3.1 Calculating Energy Values of Cones

In order to assess the number of squirrels the forest could theoretically support, it was necessary to calculate the energy values of the cones of both tree species in Derryclare. This was achieved by calculating the number of seeds in an average cone, and multiplying this by the energy value of each species' seed.

For Sitka spruce cones, the number of seeds in the average cone was calculated by performing a regression analysis on a plot showing the lengths of a random subset of cones (*n* = 110) against the number of scales per cone. The number of seeds per average cone was multiplied by the energy value of a Sitka spruce seed (0.04 kJ) (Gurnell, Lurz, and Pepper 2001) giving the total energy content per cone. For lodgepole pine cones, the scale counting method is unreliable (Benkman, Holimon, and Smith 2001). Therefore, we applied a lodgepole pine‐specific equation reported in the literature (McNab et al. 2019) to the average cone length to calculate the number of seeds in an average cone. The average cone length was calculated from a random subsample (*n* = 179). The number of seeds in an average cone was multiplied by the energy value of a lodgepole pine seed (0.098 kJ) (Gurnell, Lurz, and Pepper 2001) to obtain the energy content per cone. By multiplying the energy value of each species' average cone by their respective cone densities (the average number of cones per m^2^), the energy per m^2^ was determined. This value was then extrapolated to the mature area of the forest to obtain the estimated total energy available in the forest.

#### 2.3.2 Estimating the Potential Red Squirrel Population Size

To estimate the forest's potential squirrel population, the lower (400 kJ) and upper (700 kJ) energy requirements of a red squirrel (Gurnell, Lurz, and Pepper 2001) were utilised to calculate the minimum and maximum number of squirrels the forest could sustain. To estimate the number of squirrels present in the forest, these calculations were repeated using the densities of consumed cones and extrapolated to all mature areas, as positive squirrel signs were observed in all surveyed stands.

#### 2.3.3 Estimating Previous Potential Red Squirrel Population Sizes

The potential number of squirrels that the forest could sustain, and the estimated actual population sizes during the growth phase monitoring project (2008–2011) were estimated using feeding survey data reported by Waters (2012). These surveys were all conducted in late autumn to winter, with the exception of two additional spring and summer surveys in 2010.

The energy values of lodgepole pine cones during the growth phase were determined by applying a lodgepole pine‐specific equation to the average length of lodgepole pine cones from these years (Waters 2012), consistent with the current study's methodology. The energy values of the average Sitka spruce cone in the 2021 survey were used for the growth phase calculations, as length data were unavailable for those years. Energy values per m^2^ were then determined by multiplying the average cone's energy by their respective species' cone density, as previously described. To determine the total energy available in the forest, these values were extrapolated to the entire mature area of the forest during this phase, as determined by the digitised maps. The potential population the forest could support was calculated by dividing the total energy consumed by the energy requirements of a red squirrel, as described above. Subsequently, the total energy consumed by the squirrels was estimated by extrapolating the energy values to the actual area occupied by the squirrels at the time of each survey, as reported in the growth phase study (Waters 2012).

Finally, an alternative estimation of the potential number of red squirrels in Derryclare was conducted, based on a density of 0.32 red squirrels found in a similar lodgepole pine/Sitka spruce mix forest (Lurz and Garson 1997). This method relies solely on the total area of mature forest, rather than cone density. The carrying capacity of the population in the forest was calculated using this method for all three phases considering all available mature forest available at the time of each phase.

### 2.4 Composition Change

The examination of unpublished data provided by Waters exposed a discrepancy in the mapping, revealing that the true composition of the stands in Derryclare is not accurately reflected by the Coillte map data. Specifically, Sitka spruce was found to be more abundant than recent Coillte data suggested. This inconsistency between the map data and actual composition impacted the carrying capacity of the population of the forest, prompting an investigation in the present study to reveal the true composition of the stands during the regulation phase study (2021).

To address this issue, a sample stand with a known ratio of lodgepole pine to Sitka spruce, based on unpublished data from a visual survey by Waters, was selected. We compared the percentage composition of trees in this sample stand to the density of cones produced by each species, as recorded in the 2021 feeding survey. By calculating the ratio of cone density to tree species percentage composition, it was possible to apply this formula to the known cone densities of other stands, thereby calculating the actual tree species ratio in each respective area. The relationship is represented by the following formula, where *R*_SS1_ is the ratio of Sitka spruce to lodgepole pine in Stand 1 (where the ratio is unknown), and *R*_SS2_ is the ratio of Sitka spruce to lodgepole pine in Stand 2 (where the ratio is known). *C*_SS1_ and *C*_LP1_ represent the cone production per m^2^ for Sitka spruce and lodgepole pine in Stand 1, respectively. Similarly, *C*_SS2_ and *C*_LP2_ represent the cone production per m^2^ for Sitka spruce and lodgepole pine in Stand 2 respectively.

$$R_{\mathrm{SS}1}=\frac{C_{\mathrm{SS}1}\times C_{\mathrm{LP}2}\times R_{\mathrm{SS}2}}{C_{\mathrm{LP}1}\times C_{\mathrm{SS}2}}$$

By employing this approach, we obtained a more accurate representation of the forest's current composition. This updated composition was retrospectively used to update the 2021 map of Derryclare, as well as the carrying capacity estimation. All reported results are based on the updated composition described here, unless stated otherwise.

## 3 Results

### 3.1 Change in Habitat

**FIGURE 2:**
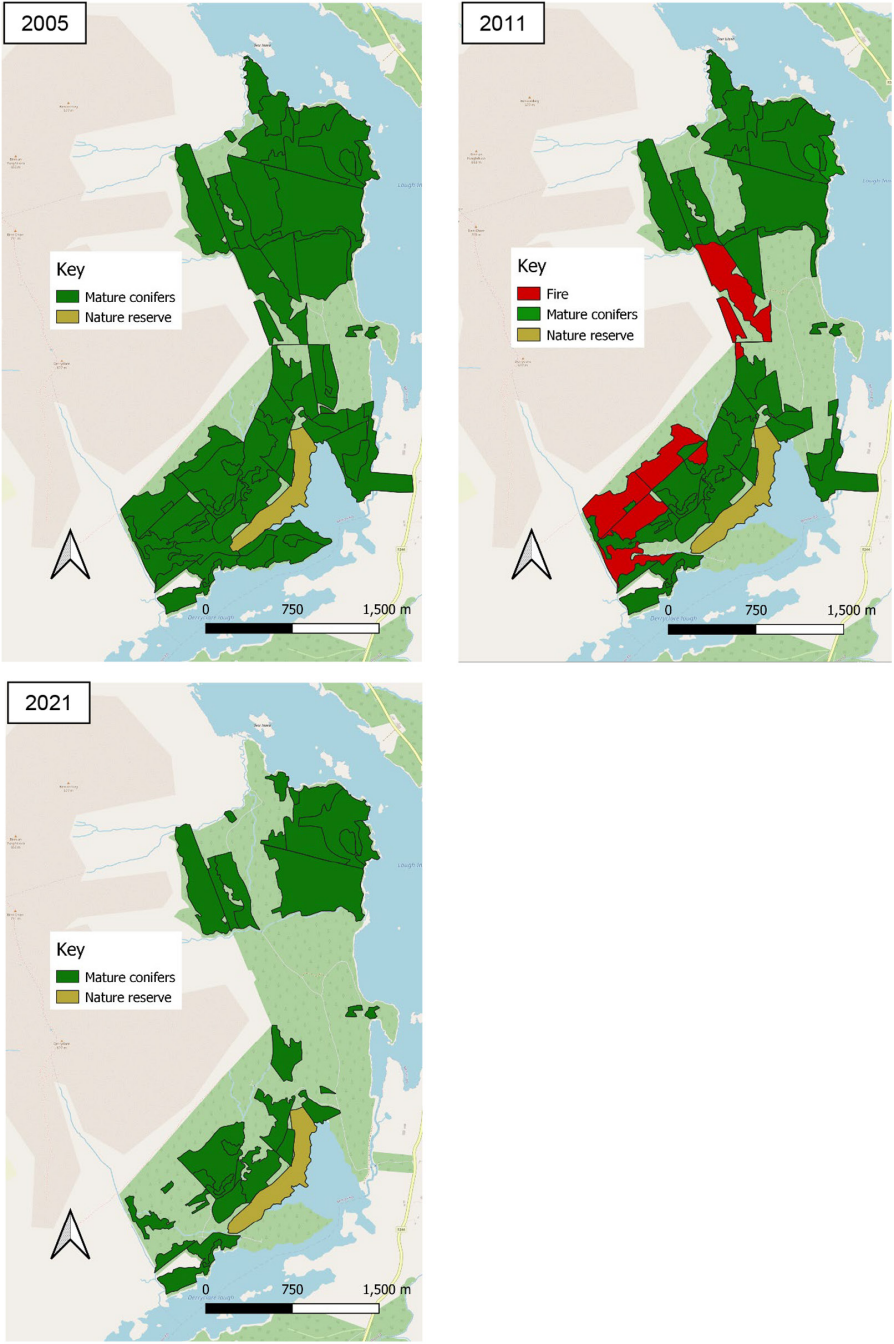
The composition of Derryclare throughout the three phases. Top left: The composition of Derryclare in 2005. Top right: The composition of Derryclare in 2011. Areas impacted by the fire of April 2011 are marked in red. Bottom left: The composition of Derryclare in 2021.

The internal structure of the forest has undergone significant changes since the introduction of the translocated red squirrels in 2005. In 2021, 202 ha were comprised of mature stands, representing a substantial reduction from the 432 ha of mature forest present in 2005. This reduction of 47% (230 ha) over 16 years was caused by clear felling (172 ha) and the fire in 2011 (58 ha) (Figure 2).

In terms of forest composition, there was a 42% reduction in the coverage of Sitka spruce, and a 60% reduction in the coverage of lodgepole pine since 2005 (Table 1). All Sitka spruce loss is due to clear felling, while 35% (58 ha) of the lodgepole pine loss was caused by the fire of April 2011. Additionally, 21% (35 ha) of the reduction in lodgepole pine coverage can be attributed to misrepresentation on the map, as described in Section 3.4.

**TABLE 1:** Composition of Derryclare forest, during the establishment (2005), growth (2008–2011) and regulation (2021) project phases, including the nature reserve (broadleaf), and immature, felled or bare stands (unsuitable).

Year	Broadleaf (ha)	Sitka spruce (ha)	Lodgepole (ha)	Mature conifers (ha)	Unsuitable (ha)
2005	13	156	276	432	138
2008	13	123	269	392	178
2009	13	108	246	354	216
2010	13	107	226	333	237
2011 (pre fire)	13	93	220	313	257
2011 (post fire)	13	93	162	255	315
2021	13	91	111	202	368

*Note:* All values are rounded to the nearest hectare.

### 3.2 The 2021 Feeding Survey

In the 2021 feeding survey, a total of 33 transects were conducted in Derryclare commercial forest, covering an area of 1650 m^2^. During the survey, 1032 lodgepole pine cones were counted, 63 of which were found to have been consumed. A total of 5599 Sitka spruce cones were counted, including 136 consumed cones. All collected Sitka spruce cones (consumed; *n* = 136, unconsumed; *n* = 215) were measured and found to have a mean length of 48.1 mm with a standard error of 0.44 (*n* = 351).

**FIGURE 3:**
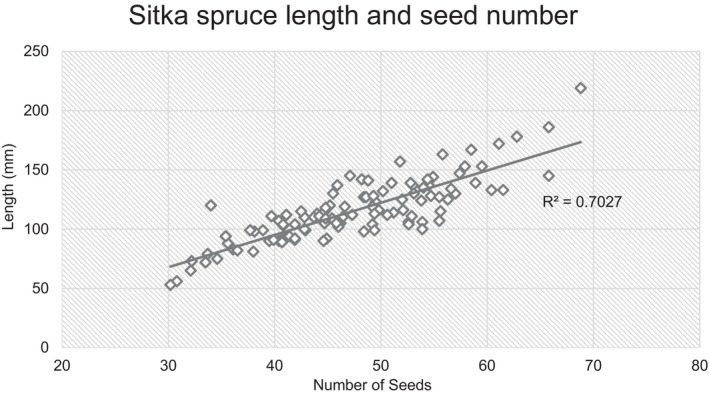
The relationship between the number of seeds per Sitka spruce cone and the cone length.

A subsample of 110 Sitka spruce cones were randomly selected and a strong positive correlation (*R* ^2^ = 0.70, *r* = 0.84, *p* < 0.001) was observed between the number of scales, and therefore seeds, and the cone length (Figure 3). The mean number of seeds in a Sitka spruce cone was calculated to be 117 with a standard error of 2.53, with an energy value of 4.69 kJ per cone.

A subsample of 179 lodgepole pine cones were measured and the average length was found to be 42.8 mm with a standard error of 0.53. The number of seeds in the average lodgepole pine cone was calculated as described above and was found to be 54 with a standard error of 0.88. The mean energy per lodgepole pine cone in this study was calculated to be 5.28 kJ.

### 3.3 Comparison With Past Feeding Surveys

The total energy available and total energy consumed during both the growth (2008–2011) and regulation phases (2021) of the project were calculated. The feeding survey conducted in 2011 occurred post‐fire (Table 2). The average number of Sitka spruce cones per m^2^ has increased eightfold from 2008 to 2021, representing a significant rise in Sitka spruce cone availability, as determined by a Mann–Whitney *U* test (*U* = 745, *n* = 64, *p* = 0.002). This increase occurred despite a reduction of 32 ha in overall Sitka spruce coverage. During this same period, Sitka spruce consumption has risen from no detection of the consumption of Sitka spruce in 2008, to the consumption of 1.99% of the energy available from Sitka spruce cones in 2021. A Mann–Whitney U test found this increase to be significant (*U* = 660, *n* = 64, *p* = 0.001). For lodgepole pine, the number of cones per m^2^ has decreased significantly compared to 2008, according to a Mann–Whitney U test (*U* = 157, *n* = 64, *p* < 0.001), exhibiting an 83% decline in cones per m^2^, while also exhibiting a reduction of 158 ha in overall lodgepole pine coverage. However, 6.34% of the available energy from lodgepole pine cones was consumed in 2021, in stark contrast to the 1.99% of Sitka spruce energy that was consumed in the same year. The total energy available in the woodland has fallen by 66% from 2008 to 2021, and the total energy consumed in squirrel occupied areas during this period has fallen by 75% (Table 2).

**TABLE 2:** Cone densities and corresponding energy values per m^2^ for both consumed and unconsumed cones of both species, during the growth and regulation phases of the project.

	Available cones								Total energy in forest (kJ)	
	Sitka spruce				Lodgepole					
Year	Cones available per m^2^	Energy per m^2^ (kJ)	Mature area (ha)	Total energy (kJ)	Cones available per m^2^	Energy per m^2^ (kJ)	Mature area (ha)	Total energy (kJ)		
**2008**	0.48	2.25	123	27,67,500	3.7	21.84	269	5,87,49,600	6,15,17,100	
**2009**	0.02	0.09	108	97,200	3.37	19.89	246	4,89,29,400	4,90,26,600	
**2010**	5.3	24.84	107	2,65,78,800	1.42	8.38	226	1,89,38,800	4,55,17,600	
**2011**	2.12	9.93	93	92,34,900	1.35	7.97	220	1,75,34,000	2,67,68,900	
**2021**	4.02	18.83	91	1,71,35,300	0.63	3.33	111	36,96,300	2,08,31,600	
	Consumed cones								Total energy consumed in forest (kJ)	% total energy
	Sitka spruce				Lodgepole					
Year	Cones consumed per m^2^	Energy per m^2^ (kJ)	Occupied area (ha)	Total energy (kJ)	Cones consumed per m^2^	Energy per m^2^ (kJ)	Occupied area (ha)	Total energy (kJ)		
**2008**	0.00	0.00	0	0	0.53	3.13	74.00	23,14,684	23,14,762	3.76
**2009**	0.00	0.00	0	0	0.71	4.19	104.00	43,57,600	43,49,882	8.87
**2010**	0.30	1.41	36	5,07,600	0.20	1.18	119.00	14,04,200	19,11,920	4.20
**2011**	0.14	0.66	64	4,19,866	0.37	2.18	126.00	27,51,417	31,71,411	11.85
**2021**	0.08	0.37	91	3,41,141	0.04	0.21	111.00	2,34,438	5,75,690	2.76

*Note:* Energy values per m^2^ were extrapolated to show total energy available in the forest. The total energy consumed was extrapolated to the areas known to be occupied by the squirrels. The percentage of the total energy available in the forest that was consumed is also shown.

### 3.4 Carrying Capacity and Population Estimate Changes

The carrying capacity of the forest's population during all three phases of the project was estimated based on the total mature forest and the total energy available. Actual population sizes were estimated based on the total energy consumed (Table 3). The estimated carrying capacity of the population, based on populations in a forest with a similar composition (Lurz and Garson 1997), has dropped by 53% since the introduction of the red squirrels to Derryclare. Additionally, the lower and upper estimates of the carrying capacity, based on the feeding survey data, show a 41%–81% decline during this period respectively.

**TABLE 3:** Changes in the carrying capacity of the population in the forest as determined by total mature forest area (rounded to the nearest ha) and feeding survey (FS) estimates.

Year	Mature conifers (ha)	Estimated carrying capacity	FS estimated carrying capacity	FS estimated actual population
2005	432	138	—	—
2008	392	125	241–421	9–16
2009	354	113	192–336	17–30
2010	333	107	178–312	7–13
2011 (pre fire)	313	100	105–183	12–22
2011 (post fire)	255	82	—	—
2021	202	65	82–143	2–4

*Note:* All carrying capacity and actual population estimates show the estimated total number of squirrels in the forest. Carrying capacity estimates based on total area use a squirrel density found in a similar forest reported in the literature (Lurz and Garson 1997), whereas the feeding survey utilises upper and lower red squirrel energy requirements to estimate the upper and lower carrying capacity values. Total population estimates based on the feeding survey results are also shown.

## 4 Discussion

The available habitat at Derryclare has declined dramatically since the introduction of the red squirrel population. The area of mature forest available to the population in 2021 is 47% of what it was in 2005. The effect of this habitat loss on the red squirrel carrying capacity in the forest has also been significant, with the carrying capacity estimate based on area declining by 53% and the estimate based on the feeding surveys declining by 41%–81%. The substantial drop in the estimated population from 12–22 in 2011 to 2–4 in 2021, as determined by the feeding surveys, reflects the negative impact of this habitat loss on the population, and likely indicates a population collapse. The observed consumption of only 2.76% of the available energy in the forest is the lowest recorded proportion in all feeding surveys conducted in Derryclare. In addition, the proportion of tree species in Derryclare has shifted over the past two decades. Lodgepole pine, which comprised 63.8% of all mature forest in 2005, has declined to 55% in 2021, the implications of which are discussed in detail below.

The translocated red squirrel population in Derryclare faced a substantial long‐term threat in the form of habitat loss, largely driven by commercial felling operations. The process of clear felling not only reduces the available habitat, but also introduces further risks for populations, both immediate and long‐term. Risks include direct mortality during the felling operations (Ewacha et al. 2017), disturbance to breeding patterns (Wilson and Wilson 1975), failure to establish new home ranges, increased predation facilitated by a lack of cover (Pausas and Parr 2018), and increased vulnerability and stress, impacting fitness (Ewacha et al. 2017). The deleterious immediate and long‐term effects of habitat loss highlight how the simplicity of estimating carrying capacity solely based on area does not capture the complexity of how changes in the environment influence populations.

In addition to habitat loss, commercial forests present several other challenges for translocations. The instability of commercial forests due to the likelihood of changes in the management's plans means that the future composition is unpredictable, making it a risky choice for a translocation. Additionally, forest plantations in Ireland traditionally consist of conifer monocultures (Iremonger et al. 2007) or develop into monocultures by planting a sacrificial ‘nurse’ conifer species alongside the primary tree species to aid its growth (Keane, Mason, and Pfeifer 2018). The low tree species diversity of these plantations makes them inherently volatile due to their vulnerability to threats such as pests and diseases (Carnol et al. 2014). It is therefore advisable to explore alternative, more stable and diverse translocation sites where possible.

The second unanticipated threat faced by the Derryclare translocated population is a deterioration in habitat quality, specifically the replacement of lodgepole pine with Sitka spruce. This is evident in the declining crop yield of lodgepole pine from 2008 to 2011, and the further drop recorded in 2021 (Table 2), in a species which is known for its stable crop yields (Benkman et al. 2003; Lurz, Garson, and Ogilvie 1998; Smith and Balda 1979). This is likely a consequence of planting lodgepole pine as a nurse species alongside Sitka spruce, a self‐thinning management practice widely employed by Coillte during the period of Derryclare's establishment, with the aim of creating stands of pure Sitka spruce (Keane, Mason, and Pfeifer 2018). However, this information was not known during the translocation planning process.

Based on a self‐thinning timescale outlined by Mason and Connolly (2018), the replacement of lodgepole pine with Sitka spruce likely began in Derryclare between 2000 and 2010. The consequences of this practice can be seen not only in the declining lodgepole pine crop production, but also in the higher proportion of Sitka spruce recorded in the forest in recent years compared to the original plantation plans (see Section 3.1). The negative impacts of this shift in composition may be seen in the decreasing amount of energy available per m^2^ in the forest. The energy provided by the more favourable and nutritious (Lurz, Garson, and Wauters 2000) lodgepole pine crop has dwindled to less than a sixth of its 2005 output. Despite this decline, lodgepole pine remains the preferred food source for squirrels in Derryclare, with 6.34% of total lodgepole energy in 2021 consumed by the squirrels, in stark contrast to the 1.99% of the total energy provided by Sitka spruce being consumed. This result aligns with the findings of Gurnell et al. (2004), who observed that Sitka spruce cones are favoured the least by red squirrels compared to other cone producing species. Therefore, the loss of lodgepole pine has disproportionately impacted the population. Additionally, the fluctuating crop mast of Sitka spruce (Broome, Hendry, and Peace 2007), as observed in the present study (Table 2), results in an unreliable food source for red squirrels. The ongoing replacement of lodgepole pine with Sitka spruce will likely have increasingly negative effects on the red squirrel population. Red squirrel population densities are negatively correlated with the proportion of Sitka spruce in a forest (Lurz, Garson, and Ogilvie 1998). While the replacement of lodgepole pine with Sitka spruce is not the sole factor contributing to the decline in carrying capacity in Derryclare, it exacerbates the issue and leads to a less favourable forest.

This further highlights that habitat size alone is not a good enough predictor of the suitability of a translocation site, and instead must incorporate site characteristics and quality (Gilpin 1986). It is crucial to investigate the specific habitat quality in relation to the target species, taking into account their ecology and behaviour (Seddon, Armstrong, and Maloney 2007). By incorporating habitat quality assessments into the selection process, the chances of translocation success can be significantly enhanced. Crucially, a habitat quality assessment must recognise that the area and quality of a habitat are dynamic and can change throughout a translocation project, as seen in Derryclare. Consequently, during the initial planning phase the habitat should not be treated as a static factor. A translocation site, like all land, may be exposed to various disturbances and alterations, including anthropogenic, natural and climate‐related factors. Therefore, a comprehensive risk assessment of potential disturbances taking into account their direct and indirect cascading effects on the target species should be conducted during the planning phase. The carrying capacity estimate in the present study based on area alone (Table 3) fails to take into account the negative effects of such changes in the environment, beyond a simple reduction in habitable area. As discussed above, habitat loss introduces a variety of new risks and challenges for the population that are not accounted for in carrying capacity estimations based solely on area. Additionally, this estimate does not consider the quality of the available area or shifts in this quality over time.

While potential changes in the habitat area and quality are important considerations to make when planning a translocation, we recognise that practical site selection often involves making concessions, as finding the perfect site is rarely possible. For example, Derryclare was chosen as a translocation site owing to its large size, age structure, accessibility, support of management bodies Coillte and the National Parks and Wildlife Service, and its distance from the range of the grey squirrel (Poole and Lawton 2009). These factors led to it being deemed the best potential site among several options that were considered in the region. The drawbacks of felling operations and a high Sitka spruce proportion were deemed to be offset by its many suitable qualities. In such cases where practical and balanced decision making results in the choice of a habitat with some suboptimal aspects, intervention may be needed to support the population in the long‐term (Grant, Johnson, and Thiessen 2019). Positive changes in the habitat have been shown to increase the chances of translocation success (Wilson 2018). Supplementary feeding is one such effort that can be employed to support the translocated population and increase the likelihood of successful establishment and long‐term regulation (Cabezas and Moreno 2007).

In conclusion, the translocation of red squirrels to Derryclare has provided valuable insights into the complexities and challenges of conservation translocations. The declining lodgepole pine crop yield and the ongoing replacement by Sitka spruce has highlighted the importance of considering habitat quality as a critical factor in translocation planning. As demonstrated in this study, habitat size alone is insufficient to ensure translocation success; instead, internal habitat characteristics and their potential changes over time should be assessed during the planning phase. We argue that, instead of hindering projects with stringent standards, a nuanced understanding of a site's quality and future serves as the foundation for well‐informed decision making. Additionally, any challenges and potential setbacks may be effectively offset by the establishment of a rigorous long‐term monitoring programme, which also allows for an assessment of the translocation's true outcome. The lessons learned from this study underscore the need for comprehensive planning, continuous monitoring, and adaptive management in translocation projects to ensure the long‐term viability and success of reintroduced populations.

## Author Contributions

**Emily Reilly:** conceptualization (equal), data curation (lead), formal analysis (lead), investigation (lead), methodology (equal), project administration (equal), writing – original draft (lead). **Colin Lawton:** conceptualization (equal), methodology (equal), project administration (equal), supervision (lead), writing – review and editing (lead).

## Conflicts of Interest

The authors declare no conflicts of interest.

## Data Availability

Data is available at the following https://doi.org/10.5281/zenodo.10610896.
